# RNA trafficking in parasitic plant systems

**DOI:** 10.3389/fpls.2012.00203

**Published:** 2012-08-22

**Authors:** Megan LeBlanc, Gunjune Kim, James H. Westwood

**Affiliations:** Department of Plant Pathology, Physiology and Weed Science, Virginia TechBlacksburg, VA, USA

**Keywords:** *Cuscuta*, haustorium, host–parasite interactions, Orobanchaceae, *Orobanche*, *Phelipanche*, RNA trafficking

## Abstract

RNA trafficking in plants contributes to local and long-distance coordination of plant development and response to the environment. However, investigations of mobile RNA identity and function are hindered by the inherent difficulty of tracing a given molecule of RNA from its cell of origin to its destination. Several methods have been used to address this problem, but all are limited to some extent by constraints associated with accurately sampling phloem sap or detecting trafficked RNA. Certain parasitic plant species form symplastic connections to their hosts and thereby provide an additional system for studying RNA trafficking. The haustorial connections of *Cuscuta* and *Phelipanche* species are similar to graft junctions in that they are able to transmit mRNAs, viral RNAs, siRNAs, and proteins from the host plants to the parasite. In contrast to other graft systems, these parasites form connections with host species that span a wide phylogenetic range, such that a high degree of nucleotide sequence divergence may exist between host and parasites and allow confident identification of most host RNAs in the parasite system. The ability to identify host RNAs in parasites, and vice versa, will facilitate genomics approaches to understanding RNA trafficking. This review discusses the nature of host–parasite connections and the potential significance of host RNAs for the parasite. Additional research on host–parasite interactions is needed to interpret results of RNA trafficking studies, but parasitic plants may provide a fascinating new perspective on RNA trafficking.

## INTRODUCTION

RNA trafficking in plants has received increasing attention in recent years. It is an important phenomenon in that it suggests that plants operate at the level of supracellular organisms, meaning that mRNA is not restricted to the cell in which it was synthesized, but rather moves from cell-to-cell and even over long distances through the phloem (Lucas and Lee, 2004). Under this model mRNAs function in cells distant from their point of origin by carrying information throughout the plant and act as part of a system for coordinating plant development.

However, the study of RNA movement between cells of a single organism presents tremendous challenges. It is difficult to confidently determine the cell of origin of an RNA molecule when the genotypes of source and destination cells are identical. This inability to distinguish mRNA origins hampers research, and while it is currently thought that there are about 1,100 proteins and hundreds of mRNAs that undergo long-distance movement (Atkins et al., 2011), the actual scope of RNA trafficking over short and long distances is unknown.

Parasitic plants are relevant to the consideration of RNA trafficking because certain species have the ability to form symplastic unions with host species wherein the connections allow the transfer of RNA. In the case of lespedeza dodder (*Cuscuta pentagona*), the connections permit transfer of mRNA from host to parasite (Roney et al., 2007; David-Schwartz et al., 2008), and this raises exciting prospects for understanding RNA trafficking in plants. Because *Cuscuta* has a relatively wide host range and can effectively parasitize a number of species from a diverse range of plant families, this parasite can act as a sink for host mobile RNA from many different species. Furthermore, the evolutionary distance between *Cuscuta* and most of its hosts means that the majority of mRNAs synthesized in a host have sequences that are divergent from those of *Cuscuta*, thus simplifying the process of bioinformatically recognizing host mRNA that has trafficked into the parasite. To the extent that *Cuscuta* connections to hosts approximate normal cell-to-cell connections within plants, *Cuscuta* can serve as an exceptionally wide heterograft to facilitate studies of mobile RNA. This review will examine the nature of host–parasite connections and consider the advantages and disadvantages of using parasites for studies of RNA trafficking in plants.

## PARASITIC PLANT CONNECTIONS: THE PERFECT GRAFT?

The connection between parasitic plants and their hosts has been compared to “the perfect graft” (Kuijt, 1983). The analogy of parasitic plant connections to graft unions is appropriate in that both involve fusing together separate plants to forge new cellular connections and vascular continuity. Both grafts and parasite connections establish symplastic connections (Although this is not true of all parasite species, it is accepted for *Cuscuta* and *Phelipanche* spp.), and have the ability to transmit RNA (Westwood et al., 2009; Harada, 2010). However, whereas man-made grafts are the result of joining cut tissues, the parasitic connection involves a highly coordinated biological invasion (Joel and Losner-Goshen, 1994; Lee, 2007). Although parasitism may elicit defense responses from the host (Borsics and Lados, 2002; Griffitts et al., 2004; Swarbrick et al., 2008), compatible reactions display little tissue necrosis and haustorial connections are characterized by close association of live cells from both species.

Another difference between graft unions and parasite connections is the greater breadth of compatibility between parasites and hosts compared to graft compatibilities. Parasites are able to form connections with plant species that are phylogenetically distant from themselves, which stands in contrast to grafting where success is greatest when stock and scion are from the same or closely related species (Mudge et al., 2009). For example, a heterograft may consist of a pepper scion on a tomato stock, but both species are members of the Solanaceae family. Parasites in contrast, commonly connect to host plants that are phylogenetically distant from themselves, with an excellent example being *Striga* *hermonthica*, a dicotyledonous members of the family Orobanchaceae that attacks grass (Poaceae) hosts. The host range of parasitic plants may also vary substantially, and some species are able to parasitize a wide range of host species while others are limited to just a single genera. Certain parasite species have adapted to attack relatively broad ranges of crop plants and are economically important agricultural weeds (Parker and Riches, 1993; Westwood et al., 2010).

Parasitic plants live by tapping into the vascular system of a host plant and withdrawing the necessary water and nutrients needed to provide part or all of the parasite nutritional needs. Parasites connect to their hosts using a specialized structure, the haustorium, which penetrates host tissue and forms a bridge to the vascular system of the host. Haustoria vary substantially in their anatomy and function among different parasitic plant species, and are generally characterized by whether they form connections exclusively to the xylem only or to both xylem and phloem (Irving and Cameron, 2009). Xylem-feeding parasites primarily withdraw water and dissolved solutes from the host and are generally hemiparasitic in that they are able to photosynthesize to produce at least part of their carbon needs. Parasites that form both xylem and phloem connections are often holoparasitic, relying on their hosts for all nutritional needs. Among this latter group are the dodders (*Cuscuta* spp.) and broomrapes (*Orobanche* and *Phelipanche* spp.), two genera with relatively well-characterized haustoria. RNA trafficking to parasitic plants has been best characterized in these species, particularly *Cuscuta*, but even here the level of understanding of RNA movement is far from complete.

With respect to *Cuscuta*, the overwhelming physiological data indicates that the parasite absorbs phloem contents from the host. However, the exact mechanism is not clear as no direct phloem connections have been demonstrated (Vaughn, 2006). Rather, cells of the parasite searching hyphae that encounter host sieve elements appear to grow around the phloem cells of the host (Dörr, 1972). These parasite cells differentiate in a manner consistent with development of sieve elements, although they also contain an elaborate network of smooth endoplasmic reticulum (ER) proximal the host cell, a feature of transfer cells (Christensen et al., 2003). For this reason it remains a formal possibility that *Cuscuta* may acquire host resources by apoplastic transfer, although this seems to fall short of explaining the ability of *Cuscuta* to readily absorb macromolecules such as mRNA, proteins, and viruses from their hosts. Physiological continuity of host and parasite phloem is sufficient to transfer the symplastic marker carboxyfluorescein within 2 h of dye being applied to the host (Birschwilks et al., 2006). This dye, as well as green fluorescent protein (GFP)-tagged viral movement protein (MP), moved readily through the phloem of established haustoria, yet was not observed extensively in host parenchyma cells outside the vascular bundle, suggesting that phloem comprises the major connection. The cell wall structure of *Cuscuta* phloic hyphae is extremely loose such that it could permit the passage of larger molecules via an apoplastic mechanism (Vaughn, 2006), but more research will be needed to definitively settle the question of phloem transfer.

In contrast to the scant anatomical evidence for direct phloem connections, *Cuscuta* has well documented plasmodesmata (PD) connections with host cells (Vaughn, 2003; Birschwilks et al., 2006). These occur along the cell walls of searching hyphae of the *Cuscuta* haustorium that traverse the host cortex to reach the host vascular tissue. The searching hyphae grow through host cells in a manner that does not puncture them, but rather forms what amounts to a tunnel through a host cell and results in the formation of new cell wall on either side, creating a chimeric structure composed of host and parasite cell walls (Vaughn, 2003). PD in these walls may take simple or branched form and are spanned by desmotubules typical of PD (Vaughn, 2003; Birschwilks et al., 2006). It is possible that these PD contribute to the transfer of materials between host and parasite, but the question has never been addressed experimentally.

The symplastic connections between broomrapes and their hosts differ from those of *Cuscuta* in terms of anatomy, but appear to share many of the same physiological functions. In contrast to *Cuscuta*, direct connections between sieve elements of *Orobanche crenata* and those of its host *Vicia narbonensis* have been imaged using electron microscopy (Dorr and Kollmann, 1995). PD between these species have also been documented and are proposed to lead to formation of sieve pores between adjacent sieve elements (Dorr and Kollmann, 1995). Anatomical differences between Convolvulaceae and Orobanchaceae parasites are not surprising given their different evolutionary origins of parasitism (Barkman et al., 2007). Broomrapes also contrast with *Cuscuta *species in the host organ targeted, as broomrapes attack roots while *Cuscuta* parasitizes stems and leaves.

## HOST–PARASITE MOVEMENT OF MACROMOLECULES

Parasitic plants acquire a wide range of macromolecules from their hosts that are relevant to the current discussion (**Table 1**). The capacity of *Cuscuta* to acquire mRNAs from hosts was first demonstrated for mRNAs of specific genes known to be mobile in pumpkin phloem (Roney et al., 2007). Total RNA was isolated from *Cuscuta* stems near the point of host attachment and used in reverse transcriptase (RT)-PCR and the resulting amplified products were then sequenced to confirm that the mRNA sequence detected in the parasite was identical to that of the host gene. Additional experiments used tomato hosts and hybridized RNA extracted from *Cuscuta* onto a tomato microarray, leading to the identification of 474 putatively mobile mRNAs. Confirmation tests on a subset of these using RT-PCR, along with an additional study (David-Schwartz et al., 2008) bring the current total of confirmed trafficked mRNAs into *Cuscuta* to 27 (Westwood et al., 2009). Mobility of mRNA into *Cuscuta* from three different hosts (alfalfa in addition to tomato and pumpkin) provides evidence that the phenomenon is not specific to just one host–parasite interaction.

**Table 1:** Macromolecules demonstrated to transfer between hosts and parasitic plants

Material	Examples	Parasite	Host(s)	Reference
mRNA	Many	*Cuscuta*	Tomato	Roney etal. (2007),
			Pumpkin	David-Schwartz etal. (2008)
			Alfalfa	
siRNA	GUS silencing	*Triphysaria*	Lettuce	Tomilov etal. (2008)
	M6PR silencing	*Phelipanche*	Tomato	Aly etal. (2009)
Viruses	Many	*Cuscuta*	Many	Hosford (1967), Birschwilks etal. (2006)
	ToMV, PVY, TYLCV, CMV	*Phelipanche*	Tomato	Gal-On etal. (2009)
Viroids	HSVd	*Cuscuta*	Cucumber	van Dorst and Peters (1974)
	PSTVd	*Phelipanche*	Tomato	Vachev etal. (2010)
Phytoplasmas	Yellows disease	*Cuscuta*	Alder to Periwinkle	Marcone etal. (1997),
			Lily to Periwinkle	Kaminska and Korbin (1999)
Protein	GFP	*Cuscuta*	Tobacco, *Arabidopsis*	Haupt etal. (2001),
				Birschwilks etal. (2006)
	GFP**	*Phelipanche*	Tomato	Aly etal. (2011)
DNA	*nad1B-C*	Rafflesiaceae	Vitaceae	Davis and Wurdack (2004)
	*atp1*, *atp6*, *matR*	*Cuscuta*	*Plantago*	Mower etal. (2004), 2010
	*atp1*	Orobanchaceae, Convolvulaceae	*Plantago*	Mower etal. (2004)
	*atp1*	*Pilostyles*	Legumes	Barkman etal. (2007)
		Rafflesiaceae	Vitaceae	
		*Cytinus*	*Helianthemum*	
		*Mitrastema*	*Fagus*	
	*rps2*	*Orobanche* and *Phelipanche*	unknown	Park etal. (2007)
	*ShContig9483*	*Striga*	grass	Yoshida etal. (2010)
	*nad1B-C*, *matR*	Santalales	*Botrychium virginianum*	Davis etal. (2005)

Host mRNAs trafficked into the parasite cover a range of biological functions that are typical of those reported in other mobile transcriptomes. For example, the list of host mobile mRNAs in *Cuscuta* include transcription factors such as the *CmNACP* and *CmWRKYP* (Ruiz-Medrano et al., 1999) and *GIBBERELLIC ACID INSENSITIVE* (GAI; Haywood et al., 2005; Huang and Yu, 2009). In addition, *Cuscuta* contains host RNAs associated with protein synthesis such as translation initiation factors and ribosomal proteins, the presence of which in phloem of other species has fueled speculation over the possibility of protein translation in sieve elements (Kragler, 2010). Finding mRNAs for genes associated with defense responses such as a cathepsin D protease inhibitor (Westwood et al., 2009; Ryan, 1990) would not be surprising given that the system is based on a pathogenic attack of one plant on another. One potential paradox is the finding of both large and small subunits of host RuBisCO mRNA in *Cuscuta* (David-Schwartz et al., 2008), which is surprising because the small subunit is generally thought to be immobile and has been used as an indicator of non-phloem contamination in studies of phloem-mobile RNAs (Ruiz-Medrano et al., 1999).

The finding of host *rbcS* mRNA in *Cuscuta* supports the hypothesis that *Cuscuta* is accessing contents of parenchyma cells as well as phloem. In an *in situ* RT-PCR experiment, mRNA of the tomato phosphofructokinase gene was shown to be concentrated in host parenchyma as well as adjacent *C. pentagona* parenchyma cells closest to the host (David-Schwartz et al., 2008). This, along with detection of transcripts localized to sieve element and companion cell regions in the parasite, suggests cell-to-cell translocation of the transcript mediated by parenchyma connections. It is possible that transcripts cross multiple layers of parenchyma cells to ultimately reach the parasite phloem, but it is also possible that the *Cuscuta* searching hyphae cells themselves differentiate into phloem or take on the role of assisting phloem cells, which would simplify the pathway to long-distance movement. Host mRNAs do move long distances as they have been found distant from the point of haustorial attachment. RT-PCR with specificity to the host transcript showed that tomato phosphofructokinase mRNA was detected in *Cuscuta* shoots up to 30 cm away from the point of contact with the host, but no target mRNA was detected beyond that point (David-Schwartz et al., 2008). This would seem to indicate a limit to mRNA translocation, although it is premature to draw any conclusions based on findings from a single gene. Additional studies are needed that characterize mobility of multiple transcripts, ideally using quantitative techniques that can reveal the dynamics of host to parasite transfer as well as long-distance movement and fate of host mRNA in the parasite.

The mobility of host mRNAs into *Cuscuta* suggests that a similar transfer would occur into broomrapes from their hosts. However this has not been demonstrated, and studies with *P. aegyptiaca* growing on pumpkin did not detect mRNA transfer even though conditions were identical to those used to demonstrate mRNA transfer into *Cuscuta* (Roney et al., 2007). More recently, data from an EST sequencing project (The Parasitic Plant Genome Project^1^) revealed no host mRNAs in the shoots of attached *P. aegyptiaca*, suggesting that host mRNA mobility into this parasite may be limited at the point of haustorial transfer into the parasite or between the tubercle and shoot of the parasite. Further experiments are needed to specifically answer this question.

Other examples of host–parasite exchange of RNA include viruses, viroids, and siRNA signals (**Table 1**). *Cuscuta* has long been recognized for the ability to accept and transmit viruses with its hosts and in fact has often been used as a vector for transmitting plant viruses between different plants (Bennet, 1944). Fifty-six viruses have thus been reported to move through *Cuscuta* (Hosford, 1967). More recent work quantified potato virus Y isolate N (PVY^N^) transmitting through a *Cuscuta* bridge between two *Nicotiana tabacum* plants, yet showed little virus accumulation in the *Cuscuta* bridge itself compared to recipient host plant and suggesting that virus movement occurs in *Cuscuta* without multiplication in the parasite (Birschwilks et al., 2006). In *P. aegyptiaca* the uptake of three positive ssRNA viruses cucumber mosaic virus (CMV), tomato mosaic virus (ToMV), potato virus Y (PVY), and the ssDNA virus tomato yellow leaf curl virus (TYLCV) were shown to move into the parasite from infected hosts and, in the case of CMV, is able to replicate in the parasite (Gal-On et al., 2009).

Viroid transport is useful to understanding long-distance RNA trafficking in general (Wang and Ding, 2010), and is interesting to consider with respect to movement into parasitic plants. Potato spindle tuber viroid (PSTVd) was shown to be taken up from a tomato host by *Phelipanche ramosa* and translocated through the tubercle and into stems of floral shoots (Vachev et al., 2010). The viroid in this study appeared to replicate in the parasite but was not mobile in the reverse direction back into the host, suggesting a dominant sinkward flow.

RNA-based silencing signals also move from host into parasite where they are able to influence gene expression. The facultative parasite *Triphysaria versicolor* (Orobanchaceae) was transformed to express the β-glucuronidase (GUS) reporter gene and then allowed to parasitize lettuce plants that expressed a fragment of the GUS gene in a hairpin orientation (Tomilov et al., 2008). Parasites attached to siRNA-expressing host plants showed a decrease in GUS staining in the root tissue near the point of host attachment, indicating transmission of the silencing signal. However, the silencing effect decreased in tissues distant from the site of attachment, suggesting that movement was somehow restricted. The signal was able to move through a *T. versicolor *bridge and into a second host plant expressing GUS. As with *Cuscuta* bridges that transmit viruses from one plant to another, it seems that parasites are able to transfer silencing signals. A similar approach was taken to silence mannose 6-phosphate reductase (M6PR) in *P. aegyptiaca* using constructs expressed in a tomato host (Aly et al., 2009). This caused a decrease in the expression of the parasite M6PR gene and raises the prospect of trans-specific gene silencing as a potential strategy for controlling parasitic weeds through engineered hosts.

In addition to RNAs, proteins have also been shown to traffic from hosts to parasitic plants. The characterization of protein mobility employed transgenic host plants expressing GFP controlled by the phloem-specific *Arabidopsis* SUC2 promoter (AtSUC2-GFP; Imlau et al., 1999). This construct was expressed in tobacco and the GFP signal translocated into *Cuscuta reflexa*, confirming the symplastic connection between host and this parasite as well with evidence of unloading of the fluorescent protein in sink tissues (Haupt et al., 2001). The same construct was used to study host protein uptake by *P. aegyptiaca* parasitizing transgenic tomato. Although an ER-targeted version of the protein did not move, a soluble version accumulated in tubercles of the parasite (Aly et al., 2011).

Parasitic plants may also acquire phytoplasmas from hosts and serve as a vector for their transmission. The leafhopper-borne yellows disease, originally believed to originate from a virus, is caused by a mycoplasma-like bacteria that flows through plant phloem (Doi et al., 1967). In two studies these bacteria, called phytoplasmas, have been shown to traverse *Cuscuta* *odorata* bridges, and infect healthy secondary *Catharanthus roseus* (periwinkle) hosts. *Cuscuta* bridges connecting Alder Yellows (ALY)-infected alder to non-infected periwinkle were able to transfer the phytoplasma, and symptoms were slowly manifested 4 months after the *Cuscuta* bridge was established (Marcone et al., 1997). In another study phytoplasmas were transmitted in 50% of the cases from *Lilium* (hybrid Casablanca) to periwinkle through the *Cuscuta* bridge, with the recipient plant developing symptoms of stunting and flower bud deficiency 2–3 weeks following connection by *Cuscuta* (Kaminska and Korbin, 1999).

The final example of host–parasite macromolecule exchange is horizontal gene transfer (HGT). Although HGT and RNA trafficking both involve movement of nucleic acids, it is not certain whether they use similar mechanisms. HGT has been reported between several non-parasitic plants, but seems to occur at a higher frequency in parasitic associations (Richardson and Palmer, 2007). The high frequency of transfer events to or from parasites is probably attributable to the greater opportunity for nucleic acid exchange provided by the close physical association of the parasitic interaction. HGT events are generally discovered during phylogenetic studies in which genes that are otherwise reliable indicators of species phylogenies instead show high homology to versions in distant families. All HGT events described to date are the result of transfers that occurred thousands of years ago, and little is known about the frequency with which HGT events occur, or the frequency at which the transferred genes are introgressed into the recipient genome.

The precise mechanism of HGT remains unresolved, but two possibilities exist. One mechanism that relates directly to the topic of mRNA trafficking is suggested by an HGT event in which a gene of unknown function moved from a grass host into *Striga* spp. (Yoshida et al., 2010). The introduced gene in *Striga* has high homology to the version in grass, but lacks introns and seems to have the remnant of a poly-A sequence, suggesting that the transfer occurred via an mRNA intermediate. In contrast, most other reported HGT events involve mitochondrial genes (e.g., *atp1*, *atp6*, and *matK*), and these have been proposed to transfer between plants as large sections of mitochondrial DNA (Mower et al., 2010). The transfer of DNA across graft junctions has been shown to occur in both directions, although the movement of DNA is limited to the region of the graft so is likely a cell-to-cell movement that does not involve phloem (Stegemann and Bock, 2009). This may be similar to what happens in parasitic plant and other natural grafts, but limited mobility of the transgenes would require development of a shoot from the graft junction to incorporate the foreign gene into the germline of the recipient plant. The study of HGT will benefit from increased sequencing of all plants, including parasitic species.

## RNA TRAFFICKING

### A MODEL FOR CELL-TO-CELL RNA TRANSPORT TO PARASITES

As a starting point, we base our understanding of host–parasite symplastic connections on what is known from cell-to-cell connections in autotrophic plants. In land plants intracellular connections need to be small enough to allow structural integrity of cell walls, while allowing for macromolecular movement throughout the plant. Cell-to-cell movement of RNA occurs through PD which are complex structures embedded in cell walls between cells and are composed of plasma membrane lined with microtubules and a continuous span of ER (Hyun et al., 2011; Burch-Smith and Zambryski, 2012). Water and solutes are able to passively travel via concentration gradients but movement is limited to molecules below 1 kDa. Facilitated movement of larger molecules is selective and usually requires localization to, and interaction with, PD (Hyun et al., 2011; Burch-Smith and Zambryski, 2012).

In order for mRNA to move in a non-cell-autonomous manner, recognition of signal sequences, or motifs, such as 3′ untranslated regions (UTRs), interact with specific RNA binding proteins much like the MPs of RNA viruses (discussed below). The MPs bind RNAs, repress translation, and enable movement of the ribonucleoprotein (RNP) complex to a PD (Baillaud, 1953; Hyun et al., 2011). Mutational studies have determined that cell-to-cell movement of RNA is also motif-dependent in order for RNPs to associate with PD trafficking proteins. Non-cell-autonomous pathway protein1 (NCAPP1) acts as a receptor for pumpkin (*Cucurbita maxima*) phloem protein 16 (CmPP16) in order to enter the phloem stream (Xoconostle-Cazares et al., 1999), and requires phosphorylation and glycosylation for entrance to PD (Lee et al., 2003). Shortroot (SHR) requires multiple motifs for mobility, but a singular specific sequence that confers mobility has not been identified (Gallagher and Benfey, 2009).

One of the best characterized mobile mRNAs is KNOTTED1 (KN1). Transcription factors of the KNOTTED1-like family are ubiquitous plant cell constituents responsible for regulating pattern formation of the apical meristem. The KN1 homeodomain protein complexed to its own mRNA was detected in phloem and was associated with determining cell fate (Lucas et al., 1995). Fluorescently labeled *E. coli* KNOTTED1 mRNA transcript was microinjected into maize mesophyll adjacent to vascular tissue, and was seen to migrate from cell-to-cell, presumably via PD connections. Mutated KN1 proteins were microinjected to show that the homeodomain protein was necessary for translocation.

**FIGURE 1:**
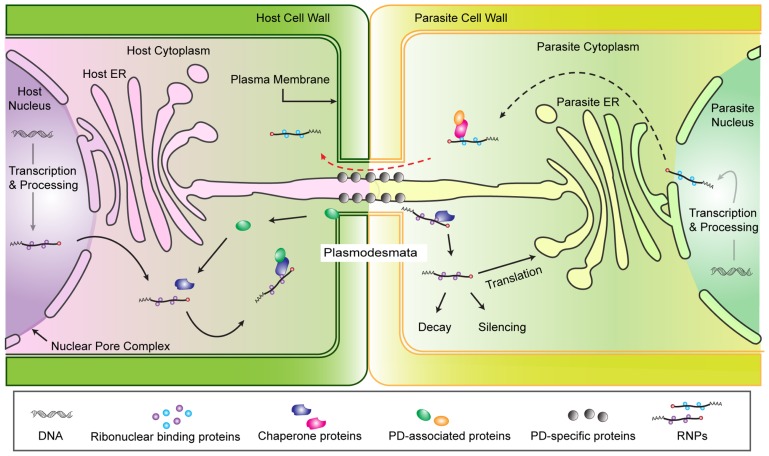
**Hypothesized model of RNA trafficking from host to *Cuscuta* via plasmodesmata.** Mature RNAs (mRNA, miRNA, or siRNA) associate with RNA binding proteins and are targeted for export from the nucleus and post-transcriptional regulation. The ribonucleoprotein complex (RNP) includes the RNA molecule plus proteins that help facilitate RNP export through the nuclear pore complex (NPC) into the cytoplasm (Lucas et al., 2001; Moore, 2005). The RNPs in the cytoplasm may be translated into protein, degraded or translocated into adjacent cells. Selective mechanisms for PD transport of non-cell-autonomous proteins (NCAPs) or RNPs () suggest chaperone proteins and/or PD-associated proteins carry NCAPs or RNPs to a PD docking protein which dilates the PD channel to transport the RNP to the cytoplasm of neighboring cells (Kim et al., 2005). Nothing is known about the fate of a host RNP after it reaches the parasite cell, but formal possibilities include translation, degradation, or modulation of parasite gene expression (e.g., silencing). The existence of similar mechanisms that would allow RNP trafficking from parasite to host are expected in parasite cells (Red dashed arrow).

Given this understanding of cellular RNA transport, we propose a model for physical interaction and RNA trafficking between host and parasite cells (**Figure 1**). We focus on host–parasite connections via a shared PD because in the case of *Cuscuta* there is both physical and experimental evidence for trafficking through PD (discussed above) and for broomrapes the PD may initiate the formation of sieve pores between species (Dorr and Kollmann, 1995). The model is based on the assumption that chimeric PD are congruent to self PD in terms of structure and function. Therefore, host RNP complexes destined for transport to another cell would associate with appropriate chaperone and PD-associated proteins and be directed through the PD. Once in the parasite cell, the fate of the RNP complex is unknown, but presumably could be processed in a manner similar to other translocated RNAs. Of course one of the interesting aspects of host parasite interactions is that the interaction is not necessarily equal and while the parasite requires an open PD for nutrient acquisition, the host would benefit by shutting down the connection. One or both plants must maintain the shared PD, with its associated plasma membrane, ER and embedded proteins, but nothing is known about this aspect of the interaction.

### VIRUS MOVEMENT AS A MODEL FOR mRNA TRAFFICKING

The mechanism of intercellular virus delivery, facilitated by MPs in cell-to-cell movement, informs our understanding of RNA transmission into and through parasitic plant attachments. Most viruses make MPs and coat proteins which assemble onto viral RNA and form viral nucleoprotein complexes (vNPCs) to facilitate passage through PD (Lough and Lucas, 2006; Lucas et al., 2009; Hyun et al., 2011). Two models demonstrate the mechanism of MP-mediated intercellular transmission of viral RNA through PD. First, the MP of a well-characterized phloem mobile pathogen, tobacco mosaic virus (TMV), may associate with ER, F-actin, and microtubules for delivery of vNPCs to PD. The MP may send a PD dilation signal to adjacent cells to allow vNPC transport (Waigmann et al., 1994; Waigmann and Zambryski, 1995; Kawakami et al., 2004), and has been shown to bind to calreticulin in the ER to aid in cytoskeletal trafficking (Chen et al., 2005). A second mechanism for virus transmission based on cell-to-cell trafficking of potato virus X (PVX) presents some of the complexities of movement of vNPCs through PD. PVX encodes three viral proteins, called a triple-gene block (TGB) that are associated with cell-to-cell movement of vNPCs to PD. TGBp2 and TGBp3 are integral endomembrane ER proteins and TGBp1 forms a TGBp1-viral RNA complex to increase SEL of PD. Anchoring the TGBp1-viral RNA complex to TGBp2 and TGBp3 mediates the delivery of the complex to the PD. In both TMV and PVX, MPs encounter SEL-binding motifs on PD proteins, which results in dilation of the channel and movement of the virus-protein complex through the PD (Haupt et al., 2005).

### LONG-DISTANCE RNA TRANSPORT

Generally, long-distance transport of RNA occurs when mRNAs from companion cells are loaded into sieve elements and are thus able to move systemically in the phloem stream. The RNP complex is stabilized as the sieve elements do not contain ribosomes for translation and lack RNAse activity that would lead to degradation, making an ideal conduit for transport of mRNAs (Kragler, 2010). mRNAs may be specifically unloaded at their destination (Haywood et al., 2005) based on a hypothesized targeting signal (or “zip code”) incorporated into the RNA or accompanying protein. Mounting evidence indicates that RNA motifs enhance long-distance trafficking, specifically the UTRs of mRNA and their associated chaperone proteins, that direct movement through the symplast. UTRs from *StBEL5* fused to the coding sequence of a less mobile BEL-homolog increased mobility (Hannapel, 2010) and the 3′ UTR of *GAI* in Arabidopsis has been shown to be required for transcript movement (Huang and Yu, 2009)

Another important example is FLOWERING LOCUS T (FT) RNA, which travels by means of a cis-acting element on the transcript, independent of the essential FT protein, from leaf to shoot apical meristem to induce flowering and suggests that mRNA has a role in systemic signaling of major developmental transitions (Corbesier et al., 2007; Li et al., 2009, 2011). If parasites form compatible cellular connections with their hosts it is possible to infer these mechanisms of RNA mobility function in much the same way in a heterologous systems, and raises questions about the potential for direct information exchange across species.

## PARASITIC PLANTS AS TOOLS FOR STUDYING RNA TRAFFICKING

Studying RNA mobility over short and long distances in plants is technically challenging because of the need to pinpoint source and destination cells of the mRNA. Techniques range from the elegant application of transgenics, phloem-sap collection (either directly or using aphid stylets), grafting, or combinations of these (Atkins et al., 2011). Each approach has advantages, but they are also subject to limitations in scope and potential for artifacts. We propose that parasitic plants can be added to this suite of techniques.

Studies of short-distance movement of mRNA have relied on transgenics and the use of genes with high specificity of expression (Kim et al., 2005), microinjection (Xoconostle-Cazares et al., 1999), or particle bombardment (Itaya et al., 1997). These approaches are excellent for demonstrating cell-to-cell movement and characterizing the features controlling trafficking of specific genes. However, these experiments are laborious and target individual genes, so are less useful for genomic-scale studies.

Investigations of long-distance trafficking are somewhat simpler because RNAs identified from phloem sap are assumed to be in the process of moving from source to destination. Direct collection of phloem sap exuded from stem incisions is the ideal, but relatively few herbaceous species are copious exuders like cucurbits (Crafts, 1932) and legumes (Sharkey and Pate, 1976). The phloem stream of most plant species is rapidly shut down by callose plugs resulting in insufficient quantities of sap. To circumvent this limitation, phloem sap can be gathered by placing the cut stem in a solution of EDTA, a chelating agent that delays formation of the calcium cation callose plug that clogs the sieve plates (Turgeon and Wolf, 2009). However, in all cases of phloem-sap collection, care must be taken to avoid contamination with cellular contents from the cut cells at the incision site. This is generally done by blotting the wound surface immediately after the incision and discarding the first several microliters of sap exudate, but this minimizes contamination rather than completely eliminating it.

Another ingenious approach for sampling phloem is aphid stylectomy, which uses the ability of aphids to precisely insert their stylets into plant sieve elements. Subsequent severing of the aphid from its stylet leaves a tube that exudes small droplets of nearly pure phloem sap (Fisher and Frame, 1984; Doering-Saad et al., 2002). Disadvantages of this method are that it is technically challenging and yields low volumes of sap, generally in the nano- or microliter range (Atkins et al., 2011). A relatively high throughput method for collecting barley phloem sap has been used that attached microcapillary tubes to the embedded stylets of 600 aphids on 30 plants, and over the course of 6 h captured 10 μl of phloem sap for protein and mRNA analysis (Gaupels et al., 2008).

Grafting is an excellent method for detecting phloem-mobile molecules where the combination of stock and scion enable differentiation of the mobile signal. For example, wide grafts (or heterografts) between related species such as pumpkin and cucumber have been used in several key studies of RNA trafficking (Lucas et al., 1995; Lee et al., 2003; Ham et al., 2009). Solanaceous species are conducive to grafting and have been used in experiments that showed that tuber formation in potato is regulated by a mobile transcription factor, *StBEL5* (Banerjee et al., 2006), and that tuber formation is increased in grafts with transgenic overexpressing scions. Where one member of the graft carries a mutation or transgene, it is possible to demonstrate trafficking of a specific mRNA and coincident transmission of a phenotype as was done for *StBEL5* in potato (Rosin et al., 2003) and the leaf shape phenotype Mouse-ears in tomato (Kim et al., 2001). A procedure for grafting together different *Arabidopsis* plants has been used for detecting flowering signals, but the success rate is relatively low compared to other graft systems at approximately 11% (Ayre and Turgeon, 2004). A disadvantage of grafting is the relatively narrow range of plants that can be joined together. Even wide grafts are restricted to members of the same plant family (Mudge et al., 2009), which leaves ambiguity in distinguishing mobile RNAs between stock and scion.

Parasites form connections to their hosts that have many of the features of grafts as well as specific advantages. Chief among these is the ability of parasites to form interspecific grafts with a wide range of species. Given the ability of parasites to form symplastic connections with many of the species commonly used for RNA trafficking studies such as *Arabidopsis* (Westwood, 2000; Birschwilks et al., 2007), tomato, and cucurbits (Roney et al., 2007), it is possible to conduct comparative studies on different plants using the parasite as a common “scion.” More importantly, the phylogenetic differences between parasites and these hosts facilitates the use of genomics approaches to understand RNA trafficking. *Cuscuta* that was grown on host species with sequenced genomes yields a mixture of host and self RNA that can be distinguish using microarrays (Roney et al., 2007) and it presents an excellent application for next-generation sequencing. Even without an extensive *Cuscuta* genome, the host sequences can be determined for most genes based on exact matches to known host sequences. An extensive database for expressed genes of Orobanchaceae parasites exists (Westwood et al., 2012), which will improve confidence in distinguishing host and parasite RNAs. The broad host range of *Cuscuta* will also facilitate studies on species that do not graft well or from which phloem exudates are difficult to obtain.

A concern over analyses of phloem sap obtained from incisions is that the sudden release in pressure would create artifacts such as dislodged macromolecules that would otherwise not be mobile in sieve elements (Atkins et al., 2011). Parasitic plants avoid this concern because the process of parasitism unfolds gradually, so presumably is accomplished without the artifacts arising from sudden pressure changes. Of course the parasite creates a strong sink that draws material from the host, but with negligible leaf surface area (for *Cuscuta* and *Phelipanche*), the process of withdrawing host vascular contents is likely within the range of normal plant translocation.

As with other methods of studying RNA trafficking, host-*Cuscuta* connections have certain disadvantages. Among these are the technical issues of establishing connections on specific host locations and at specific times. Parasites are somewhat unpredictable, and generating uniform tissues from synchronized attachment points can be challenging. In *Cuscuta*, the haustoria also follow a developmental progression through stages of host penetration, vascular connection, and eventually occlusion (Vaughn, 2003), and although the period of open transfer of macromolecules likely spans many days or weeks, there is little information on how to distinguish actively translocating haustoria from those that are too young or old to function well. Thus, the most important limitation may well be the dearth of information on the precise functioning of haustorial connections. Finally, the interaction of host and parasite is ultimately one of pathogen and host, and it is reasonable to expect that control of the PD and macromolecule exchange is a point of contention between the two species. One consequence of this is that the mRNAs trafficked from host to parasite will be enriched in pathogen/defense response functions, but this is also true to some degree for all other methods for sampling phloem contents.

## POTENTIAL SIGNIFICANCE OF TRANS-SPECIES RNA TRAFFICKING

The most intriguing questions regarding cross species movement of RNA are whether and how RNA from one species functions in another species. Answering this question is difficult because mechanisms of action of native trafficked RNAs are not fully understood. If mRNAs function through translation into protein, as has been indicated for *KN1* (Kim et al., 2005), then parasites should be able to process host transcripts as well as they process their own mobile mRNA (**Figure 1**). On the other hand, if trafficked RNA acts in a sequence-specific manner, then function in a parasite would depend on the presence of sufficiently homologous genes in the parasite. Many trafficked mRNA may not have a metabolic or regulatory function and may simply serve as a nutrient source for the parasite. Of course all three of these possibilities may occur, depending on the transcript in question.

Mobile RNAs and proteins have been shown to influence leaf shape (Kim et al., 2001; Haywood et al., 2005), tuberization (Banerjee et al., 2006), and flowering (Li et al., 2011). There is little evidence for transmission of a host phenotype in parasites, as the general morphology of the parasite does not depend on which host is used. Of course, parasites may be deficient in key regulatory pathways, for example, those that lack expanded leaves would not be expected to perceive and respond to altered signals for leaf shape. A particularly intriguing area is flowering, and some authors have suggested that *Cuscuta* flowering time depends on timing of host flowering, thus invoking the possibility that *FT* RNA and protein from the host are capable of inducing flowering in the parasite (Fratianne, 1965; Corbesier et al., 2007). If the trafficked mRNA is a transcription factor, it is possible that the delivery of just a few molecules to the right cells could effectively throw the switch to turn on a new developmental program (Kragler, 2010).

From an evolutionary standpoint, it is reasonable to think that parasites would be under selective pressure to decode and use information from host mRNAs and proteins that reveal the host physiological status, thereby enabling the parasite to respond to any changes in the host system. For example, by recognizing when the host is preparing to flower or senesce, the parasite can complete its own reproductive cycle in time. Considering that the host forms a dominant feature of the parasite’s environment, it would not be surprising if the parasites were found to have mechanisms to monitor the health and developmental status of their hosts.

One mechanism for communication that has empirical support is post transcriptional gene silencing. The ability to silence GUS in transgenic *Triphysaria* and M6PR in *Phelipanche* indicates that the process works across species as long as the RNAi construct matches the target gene in the parasite. We have no information to date on whether parasites contain homologs of silencing targets known from other plant species, but we hypothesize that parasites may encounter miRNA signaling molecules that are generated in stressed plants (Lu and Huang, 2008; Buhtz et al., 2010). Of the many identified miRNAs, miRNA399, a phosphate starvation response signal, has been shown to move into grafted tissues (Buhtz et al., 2010), so likely passes into parasites. It will be interesting to learn whether parasites have sufficiently conserved homologs to be affected by this type of regulation. For perspective we asked this question of four *C. pentagona* genes with sequences available in the 1KP Project^2^. Genes for a pentatricopeptide repeat-containing protein (Solyc01g081290.2.1), DNA mismatch repair protein (Solyc02g082660.2.1), clathrin heavy chain (Solyc06g051310.2.1), and ATPase subunit 1 (Solyc11g039980.1.1) are 80% (464/583), 83% (166/199), 85% (720/852), and 96% (251/261) identical, respectively, at the nucleotide level between tomato and *C. pentagona *(Bombarely et al., 2011). This suggests that for certain genes it is likely that a highly homologous 21 nucleotide silencing signal, such as miRNA, could act between host and parasite.

Because parasites are foremost feeding on their hosts, it is possible that the RNA is taken up as a nutrient and carried with the bulk movement of solutes from the host plant with no informational significance for the parasite. The host trafficked mRNA in parasites may undergo catabolism to provide inorganic phosphate and sugars for conservation of cellular and organismal homeostasis of the parasite or to maintain haustorial function. However, for this to be true, the parasite must have a mechanism to distinguish host trafficked RNA from its own. This might be possible given sufficient differences between host and parasite RNA and protein sequences if the limitation on function was at the level of uptake into parasite cells. By this hypothesis, parasite PD would discriminate and only allow uptake of self RNPs. However, if such precise recognition is possible, it would suggest that the parasite ability to differentiate RNPs would decrease when parasitizing closely related species, or for certain highly conserved genes. No evidence exists that parasite host ranges are specifically aimed at targeting the most distantly related species.

There may be an evolutionary benefit to trafficking of nucleic acids into the parasite, such as for horizontal gene transfer (Yoshida et al., 2010). Many parasites lineage including Rafflesiaceae, Orobanchaceae, *Cuscuta,* Mitrastemonaceae, Santalales, and *Pilostyles* (Apodanthaceae) show evidence of HGT events in phylogenetic studies (**Table 1**). However, the functional significance of most HGT events involving parasites is unclear and a benefit to the parasite has not yet been demonstrated for any gene acquired in this way.

Finally, it is interesting to speculate whether RNAs from the parasite could be used as pathogenic factors in establishing and maintaining host connections. Although an assay for parasite-to-host movement of a *C. pentagona* *PYROPHOSPHATE-DEPENDENT PHOSPHOFRUCTOKINASE* β subunit mRNA was negative (David-Schwartz et al., 2008), the movement of viruses and RNAi signals through parasite bridges from one host into another suggests that bidirectional movement of mRNA is also possible (Birschwilks et al., 2006; Tomilov et al., 2008). If RNA trafficking is important in cell-to-cell communication, it would be reasonable to expect that parasites have evolved a way to use this system to their advantage.

## CONCLUSIONS AND FUTURE DIRECTIONS

Parasitic plants that form symplastic connections with their hosts provide a new perspective on cell-to-cell and long-distance trafficking of RNAs. The haustorium creates a union with the host that resembles a graft in many ways, but is established through invasive growth of the parasite and is coordinated with host cells and therefore presents a unique type of junction. Macromolecules including RNAs, proteins, and DNAs, are able to traffic between the plants and the system presents new opportunities for studying RNA movement. Parasitic plants are ultimately pathogens, and the interactions may not be precisely equivalent to the connections between two cells of the same plant, and it is almost certain that parasitic plants have adapted the normal structure and function of PD and RNA trafficking machinery to meet their needs. Unraveling the intricacies of the parasite–host interface is likely to contribute to understanding these phenomena in the same way that studies of viruses hijacking the RNA trafficking system played a major role in elucidating components of PD function (Carrington et al., 1996). The use of parasitic plants for understanding RNA trafficking will benefit from deeper understanding of haustorial function. The interface between two plants involves coordination at many levels, from structural (shared PD) to signaling. Further research on this interaction will provide new insights into cell–cell interactions

An important feature of the haustorial connection is the ability to connect to diverse host species and this leads to a unique system in which the mobile transcriptome of one species is mixed into that of another. The situation raises intriguing questions about the functions of RNA trafficking in plants. Which RNAs are trafficked, are they targeted specifically, and how do they function at their destination? Are they translated into protein, do they modify gene expression, or are they recycled into raw material for nutrition of the recipient cells? Many of these questions can be addressed using parasitic plants. The ability to readily distinguish host and parasite sequences facilitates identification of mobile transcripts and their movement and fate in the parasite can be tracked using quantitative methods. Next generation sequencing provides the power to reconstruct mobile transcriptomes of hosts from parasite-derived RNA populations.

## Conflict of Interest Statement

The authors declare that the research was conducted in the absence of any commercial or financial relationships that could be construed as a potential conflict of interest.

## Footnotes

1 http://ppgp.huck.psu.edu/

2 http://www.onekp.com
